# SNP-Target Genes Interaction Perturbing the Cancer Risk in the Post-GWAS

**DOI:** 10.3390/cancers14225636

**Published:** 2022-11-17

**Authors:** Wenmin Yang, Te Zhang, Xuming Song, Gaochao Dong, Lin Xu, Feng Jiang

**Affiliations:** 1 Department of Thoracic Surgery, Nanjing Medical University Affiliated Cancer Hospital and Jiangsu Cancer Hospital and Jiangsu Institute of Cancer Research, Nanjing 210009, China; 2 Jiangsu Key Laboratory of Molecular and Translational Cancer Research, Cancer Institute of Jiangsu Province, Nanjing 210009, China; 3 The Fourth Clinical College, Nanjing Medical University, Nanjing 210009, China; 4 Collaborative Innovation Center for Cancer Personalized Medicine, Nanjing Medical University, Nanjing 211116, China

**Keywords:** genome-wide association analysis, single nucleotide polymorphism, cancer, molecular and biological mechanism

## Abstract

**Simple Summary:**

Genome-wide association studies have identified a vast number of cancer risk-associated loci harboring numerous single nucleotide polymorphisms that regulate gene expression and affect individual genetic susceptibility to cancer through different routes. Recently, there has been some progress made regarding the molecular and biological mechanisms underlying the ways that genetic variation affects gene regulation. This review summarizes the molecular and biological mechanisms of genetic variation that affect gene regulation by drawing from the findings provided by past studies.

**Abstract:**

Cancer ranks as the second leading cause of death worldwide, and, being a genetic disease, it is highly heritable. Over the past few decades, genome-wide association studies (GWAS) have identified many risk-associated loci harboring hundreds of single nucleotide polymorphisms (SNPs). Some of these cancer-associated SNPs have been revealed as causal, and the functional characterization of the mechanisms underlying the cancer risk association has been illuminated in some instances. In this review, based on the different positions of SNPs and their modes of action, we discuss the mechanisms underlying how SNPs regulate the expression of target genes to consequently affect tumorigenesis and the development of cancer.

## 1. Introduction

Cancer is a primary cause of mortality and a major impediment to improving life expectancy in every country on the planet [1,2]. According to the latest cancer burden data released by the World Health Organization’s International Agency for Research on Cancer (IARC), 19.3 million new cancers were diagnosed worldwide in 2020, resulting in nearly 10 million deaths, and it is predicted that over the next two decades, as populations age, unhealthy behaviors increase, and other factors influence, the number of cancer cases worldwide could increase by 60% [1]. In low or middle-income countries, the increase could be as high as 81%. In 2020, breast cancer surpassed lung cancer to become the most commonly diagnosed cancer, with an estimated 2.3 million new cases (11.7%); however, lung cancer still continues to be a major cause of mortality, accounting for an estimated 1.8 million deaths (18%) [1]. Modifiable risk factors account for more than 40% of cancer incidence and mortality in China, including tobacco exposure, obesity, unhealthy lifestyle and alcohol consumption. [3]. In addition, epidemiological research strongly suggests that the etiology of common malignancies is hereditary. Elevated cancer incidence in affected families is attributable to a complex etiology, whereby several low-penetrance cancer predisposition genes interact with environmental variables to affect cancer risk [4,5].

In recent years, genome-wide association studies (GWAS) have emerged as an effective means to determine genetic contributions to cancer risk. GWAS provide an agnostic approach to the identification of genetic variations influencing cancer risk. GWAS research, as opposed to typical candidate-driven studies, consider the full genomic sequence obtained from a large sample of cancer patients, and then compare the data to those of a healthy population, thereby allowing for a more objective approach to identifying genetic aberrances in cancer. GWAS have been performed for most types of cancers, and hundreds of risk alleles have consequently been identified, most of which are common and individually confer a modest increase in cancer risk [6].

Single nucleotide polymorphism (SNP) is a DNA sequence polymorphism caused by a single nucleotide variation at the genomic level, which refers to a series of single nucleotide changes in DNA [7]. SNP is a variation in which more than 1% of a population does not carry the same nucleotide at a specific position in the DNA sequence. Furthermore, SNP is non-pathogenic, or not directly responsible [7,8]. However, mutation is an irreversible sequence variation in DNA, which in essence includes all variations that occur in the human genome either spontaneously or non-spontaneously [9]. The frequency of mutations is relatively low, generally less than 1%, and most mutations are pathogenic. The distribution of SNPs and mutations is region-specific and race-specific. 

Multiple GWAS have been conducted during the past decade for each of the main types of malignancies, such as prostate [10,11,12], breast [13,14,15], colorectal [16,17,18,19], lung [20,21,22,23,24], gastric [25,26,27,28], renal [29,30,31], and ovarian [32,33] cancers. GWAS for thyroid cancer [34], glioma [35,35], and malignant melanoma [36,37,38] have also been reported. Currently, more than 430 cancer associations in 262 distinct genomic regions have been identified by GWAS, and increasingly more loci are being identified over time. However, due to the existence of linkage disequilibrium and the complex interactions that exist between genes and the environment, tag SNPs identified by GWAS are not necessarily true pathogenic variants. Therefore, it is important to interpret GWAS results with greater scrutiny. Freedman and Edwards et al. raised the point that post-GWAS research strategies aimed at screening functional sites and elucidating their underlying molecular mechanisms [39,40]. Up until now, there have been numerous studies conducted on the functional resolution of cancer risk SNPs. Herein, to better understand the role of functional SNPs in tumorigenesis and cancer development, we propose several working classifications for function SNPs based on region, and elaborate the mechanisms that tumor risk-associated SNP complex regulated target genes.

## 2. Functional Mechanisms of Coding Region SNPs

SNPs located in coding regions can be divided into two types: synonymous and non-synonymous mutations. Although synonymous mutations do not affect the amino acid sequence of the protein, they may change the expression of the protein by affecting post-transcriptional modifications, translation rates, and other processes. In contrast, non-synonymous SNPs (nsSNPs) cause the substitution of amino acids, thereby resulting in changes to the protein structure, its physical and chemical properties (stability, solubility, etc.), and its function. At present, there are many biological software packages (such as SIFT(Sorting Intolerant From Tolerant), F-SNP(the Functional Single Nucleotide Polymorphism) and PolyPhen) that can be used to predict the effect of nsSNPs on protein structure and function [41,42,43]. Compared to SNPs located in gene non-coding regions, the functional mechanism underlying tumor-associated nsSNPs is relatively simple [44]. Combined with whole exon analysis, several coding region SNPs have been identified to be associated with colorectal cancer development. For example, the missense mutation rs3184504 (p. trp263ARg) located in a domain of SH2B3 may change the function of the protein in the context of regulating cell division. Other coding variants may also affect variable shear (RS16888728, UTP23) [45]. The mechanism by which SNPs located within the coding regions of genes affect the risk of disease is inseparable from the function of the resulting coded proteins. 

Some risk loci exert an effect on the amino acid sequence of the produced protein. Examples include BRCA2 p.Lys3326Ter (rs11571833), and CHEK2 p.Ile157Thr (rs17879961) in lung [46] and breast [47] cancers. The mechanistic interpretation of such variants is presumed to be relatively simple. In addition to the aforesaid, coding SNPs can affect RNA processing; an example is rs78378222 in the 3′ untranslated region of TP53, whereby the risk-corrected variation alters the sequence AATAAA to AATACA, thereby changing the polyadenylation signal of TP53, and ultimately resulting in the impaired 3′-end processing of TP53 mRNA [48,49]. Some variants can also affect splicing. Tian and his colleagues identified a single-nucleotide variation in the *ELP2* gene that affect *ELP2* exon pre-mRNA splicing through splicing a quantitative trait locus (sQTL) [50].

Researchers have often focused on specific signaling pathways, genes, and genetic modifications of interest, while also performing whole-exon association analysis (GWAS) to find any relevant coding SNPs with large effects on these molecules and processes. For example, Li and colleagues used exon sequencing and conducted an association analysis of 12 important genes involved in TGF-β signaling to find that low-frequency causative variation in the TGF-β pathway contributes to colorectal carcinoma (CRC) susceptibility. They discovered that the missense variation rs3764482 (c. 83C>T; p. S28F) located in the gene *SMAD7* was consistently and strongly associated with CRC risk. The rs3764482 allele T was more effective compared to the dominant allele C in limiting TGF-β signaling and reducing the phosphorylation of receptor-regulated SMADs (R-SMADs) via impeding the activation of downstream genes, thereby promoting cancer cell proliferation and contributing to CRC pathogenesis [51]. 

**Figure 1:**
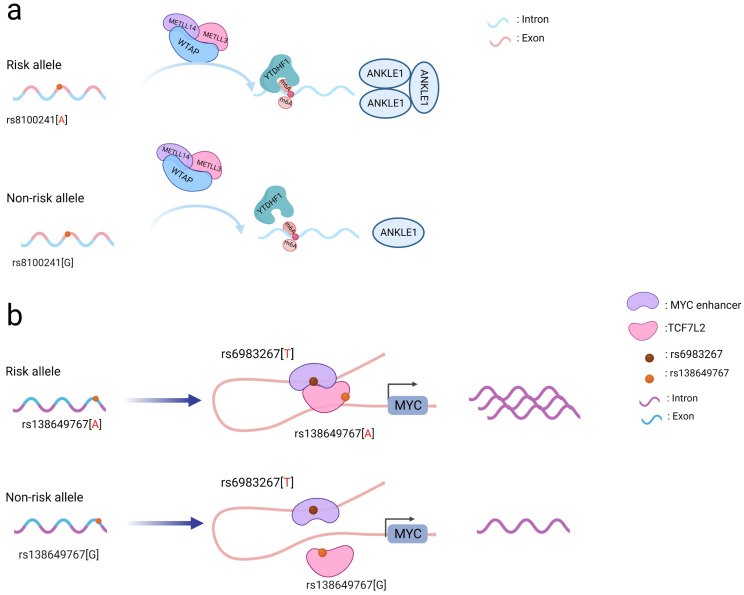
Schematic diagram of the action mechanism employed by coding SNPs. (**a**) The A allele of the rs8100241 variant, which is found in the *ANKLE1* second exon region, has been linked to a lower risk of CRC by increasing *ANKLE1* mRNA m6A levels and thus facilitating ANKLE1 protein expression, thereby potentially functioning as a negative regulator to hinder cell growth by maintaining genomic stability. (**b**) Interaction between the *TCF7L2* missense variant rs138649767 and a regulatory variant rs6983267 in the *MYC* enhancer and promoter on the expression of MYC.

Coding SNPs may also affect gene and protein modifications. The N6-methyladenosine (m6A) modification is critical for ensuring messenger RNA stability and is involved in many biological activities, including pre-mRNA splicing, 3’-end processing, nuclear export, translation regulation, mRNA degradation, and the DNA damage response [52,53]. The m6A methylation modification occurs in the messenger RNA(mRNA) and can be formed by methylation “writers” and removed by demethylation “erasers” [54]. Rs8100241, located in the gene *ANKLE1*, was identified to be associated with susceptibility to both CRC and breast cancer. The presence of the rs8100241 risk allele A (Figure 1a) combined with the m6A “writer” complex (comprised of the proteins METTL3, METTL14, and WTAP) and the m6A “reader” protein (YTHDF1) was found to increase the levels of the m6A modification on the gene *ANKLE1* and consequently increase its protein expression. Mechanistically, ANKLE1 functions as a potential tumor suppressor by decreasing CRC cell proliferation while maintaining genomic integrity, thereby contributing to a lower risk of CRC [55].

Notably, coding SNPs may interact with other SNPs to produce a stronger functional role [45]. The rs138649767 A allele (Figure 1b) located in the exon region of *TCF7L2* can activate the *MYC* enhancer containing rs6983267 allele G to promote the expression of MYC [56]. SNPs occurring in the exons and introns of *SMAD7* may affect its regulation and jointly affect downstream signaling pathways involving SMAD7 and TGFβ [51]. As a result, while examining SNPs in coding regions, the interactions between them should be taken into account to better understand their functional processes.

## 3. Functional Mechanisms of Non-Coding Region SNPs

Accumulating evidence shows that a SNP in non-coding regions is the most common type of genetic variation in the human genome, accounting for 90% of inter-individual variation [6,57]. Depending on the location, the region can harbor a response element that is either proximal (promoter, enhancer, or super-enhancer) or distal (intergenic or intra-genic). The risk loci identified by GWAS were located in the genomic regions of cell type-specific active chromatin, and most of them were quantitative trait loci, methylation quantitative trait loci and transcription factor (TF) binding related loci. Chromatin conformational studies have helped to link regulatory regions localized by SNPs to their respective target genes [6,58,59]. These loci may be involved in gene transcription, post-transcriptional processing, translation, post-translational modifications, and other processes to regulate gene expression. Many target genes have been identified using expression quantitative trait loci (eQTL) to detect the relationship between SNPs and gene expression. Non-coding SNPs can regulate the transcription of target genes by sequence-proximal (*cis*)- or distal (*trans*)-interactions. Studies have found that histone modifications in the regions of such risk SNPs are particularly abundant, especially those related to promoter and enhancer activities (H3K4me3, H3K4me1, H3K27ac). Most SNPs are predicted to destroy the binding motifs of specific transcription factors. For example, rs6983267 may change the binding of transcription factors such as MYC, CTCF, and TCF7L2 [16]. In addition to affecting gene transcription levels by altering transcription factor-binding sites (TFBS), non-coding SNPs also change epigenetic modifications and/or the chromatin structure to influence target gene expression. Through the above method, non-coding SNP participates in cell proliferation, apoptosis, migration, and invasion.

### 3.1. Genetic Variants That Alter Promoters

**Figure 2:**
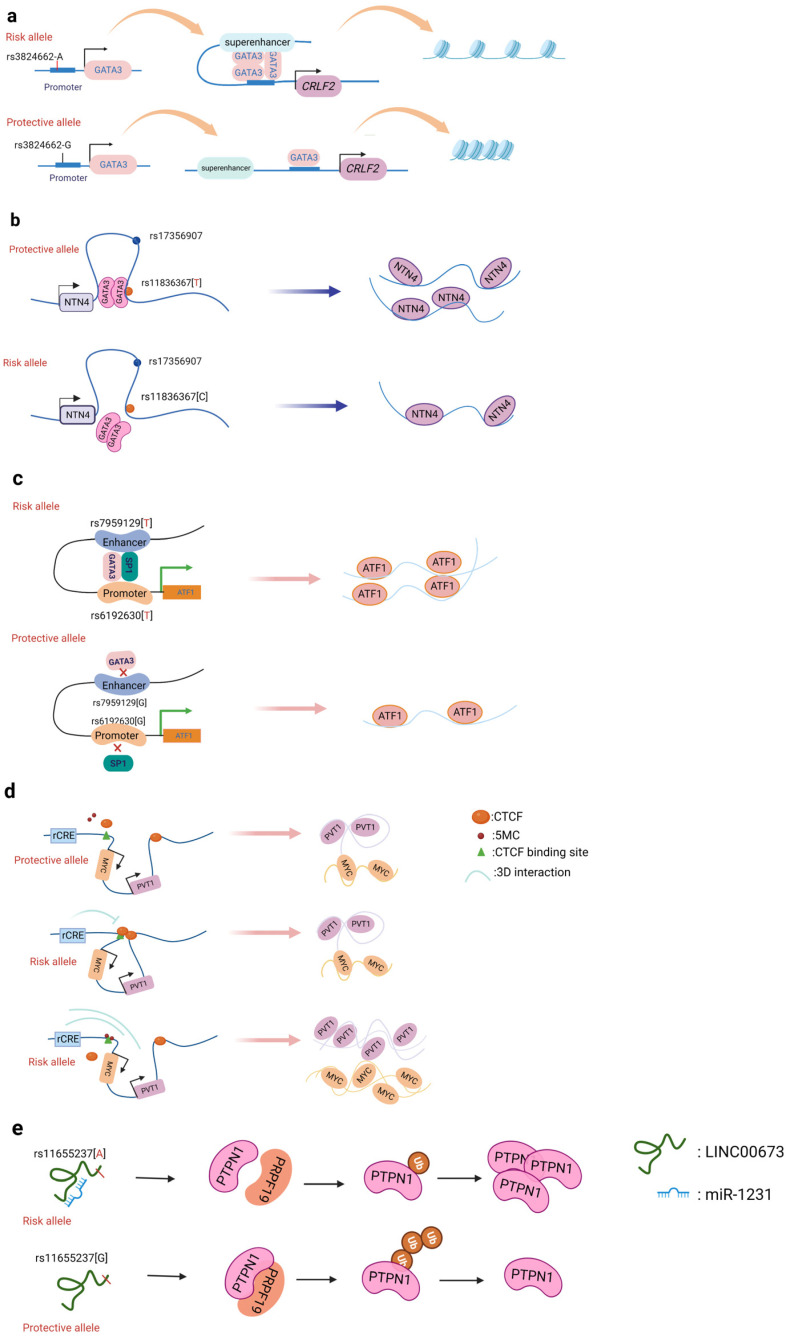
Schematic diagram of the action mechanism employed by non-coding SNPs. (**a**) The SNP rs3824662 allele A increases chromatin accessibility by inducing GATA3 expression, promoting the binding of GATA3 with the CRLF promoter, and ultimately forming a chromatin loop. (**b**) The NTN4 enhancer risk variant rs11836367 binds to the TF GATA3 to regulate NTN4 expression, ultimately promoting breast carcinoma initiation and progression. (**c**) Enhancer SNP rs7959129 risk allele G interacts with promoter SNP rs6192603 risk allele G contributing to ATF1 expression by binding TFs GATA3 and SP1. (**d**) The risk allele rs11986220 and higher methylation at –10 Kb synergistically function to confer a greater risk of tumor; however, when −20 Kb is hypomethylated, the function of the risk SNP is inhibited by the enhancer-blocking insulator loop mediated by CTCF. (**e**) The risk variant rs11655237 in LINC00673 creates a miR-1231–binding site that interferes with the expression of LINC00673 and contributes to pancreatic cancer susceptibility.

A promoter is a sequence of DNA that is recognized, bound, and serves to initiate transcription by RNA polymerase. Promoters contain variations of a conserved sequence required for the specific binding of RNA polymerase and transcription initiation. Most promoters are located upstream of the transcription initiation point of structural genes, and the promoter itself is not transcribed [60]. Promoters are located upstream of the 5’ end of a given structural gene, and they activate RNA polymerase to bind accurately to the template DNA with specificity for inducing the initiation of transcription [60]. Promoters do not control gene activity themselves; rather, gene activity is regulated by binding to proteins called transcription factors (TF). SNPs within promoter regions generally play a regulatory role by influencing the binding of such transcription factors. A recently reported example is that of the SNP rs13278062 located in the promoter of death receptor 4 (DR4) which confers an altered risk of colorectal cancer. The study revealed that the rs13278062 G>T variant changed the binding affinity of the transcription factor Sp1/NF1, increased the expression of DR4, and thus suppressed carcinogenesis and metastasis of colorectal cancer [61]. The MPO promoter SNP rs2333227 increases the malignant characteristics of colorectal cancer by changing the promoter’s affinity to AP-2α [62]. The variant SNP rs10993994 located in the upstream promoter of the gene MSMB is also found to be overrepresented in individuals with prostate cancer; this is attributed to stronger CREB binding and thus increased promoter activity [63]. Furthermore, the SNP rs11672691 is a risk locus associated with prostate cancer that is related to the lncRNA PCAT19. The non-risk variant rs11672691 and its linkage disequilibrium (LD) SNP rs887391 are more likely to bind the TFs NKX3.1 and YY1 to the PCAT19-short promoter, thereby leading to increased promoter but lower enhancer activity, which then activates PCAT19-short, and ultimately results in lower prostate cancer susceptibility [64]. SNPs in promoter regions of multiple genes, including TERT, KLHDC7A, PIDD1, and ESR1, have been discovered in breast cancer by GWAS, with reporter studies revealing that independent risk alleles change target promoter activity [65,66]. Most of the reported promoter changes exert their regulatory effects by altering TF binding. The SNP rs3824662 allele A (Figure 2a) increases chromatin accessibility by changing the TF GATA3 expression, promoting the binding of GATA3 with the CRLF promoter, and ultimately forming a chromatin loop [67].

### 3.2. Genetic Variants That Alter Enhancers

Enhancers are regions of DNA sequence that can increase the *cis*-acting transcription of their target gene sequences. Enhancers each differ in their distance from their target promoter(s); in mammalian species, an enhancer can be 100 bp to Mb away from their target gene [68]. Enhancers, unlike promoters, can be found anywhere in a gene; they can be positioned either upstream or downstream of their target genes, or even within another gene’s gene body, and enhancer regulation can circumvent other genes irrespective of their orientation. Enhancers must bind to specific protein factors to enhance the transcription of their target. Enhancers generally have tissue or cell specificity, whereby they only show activity in certain cells or tissues, which is determined by the specific protein factors present in these cells or tissues [69]. Enhancers are typically recognized by the epigenetic marks H3K4me1 and H3K27ac, which are present in active enhancer elements. Conversely, H3K27me3 is regarded as a silent epigenetic mark associated with lower enhancer activity [70,71]. GWAS-identified risk loci for common illnesses are often found in non-coding areas, and many of these are thought to function as enhancers [72]. According to emerging data, these SNPs may influence gene regulation by changing the binding of important TFs to critical transcriptional enhancers [73].

#### 3.2.1. Breast Cancer

Of all cancers, breast cancer has so far yielded the greatest number of discovered risk loci [13]. Understanding the driving mechanism(s) underlying malignant transformation provides the prospect of combating cancer recurrence and treatment resistance. Zhang et al. identified that the SNP rs4971059 resides in the sixth intron and within an active enhancer element of the TRIM46 gene. By using CRISPR/Cas9-mediated homologous recombination, they constructed the SNP rs4971059 with the allele G converted to allele A, thereby resulting in TRIM46 overexpression, boosting breast carcinoma cell growth, enhancing chemotherapy resistance in vitro, and hastening tumor development in vivo [74]. In addition, Yang and colleagues (Figure 2b) reported the noncoding regulatory variant rs11836367 at the NTN4 locus (12q22) and identified it to be associated with the risk of breast carcinoma as a causal variant. The rs11837367 protective T allele promotes GATA3 binding to the distal enhancer and increases NTN4 expression [75].

#### 3.2.2. Prostate Cancer

Several studies have independently identified several genes in specific prostate cancer (PCa) susceptibility loci that are either controlled by causative SNPs containing a *cis*-regulatory element (CRE) or have been indicated as SNP-associated genes [76]. SNP rs339331 at 6q22 was found to be a prostate cancer risk-associated variant. The risk allele T of rs339331 has been found to augment the enhancer-binding of HOXB13, alter the level of the RFX6 protein in an allele-specific manner, and confer a predisposition to prostate cancer [77]. Recently, Huang et al. also identified that the PCa-associated rs11672691 located within an enhancer element can change the binding site of HOXA2, which in turn promotes oncogenesis by impacting the expression of nearby genes [78]. 

Notably, there are other cases of SNPs causing DNA-binding polymorphisms in distinct transcription factors. For example, a gastric cancer risk-associated polymorphism (rs2978980 T>G) that is situated in an intronic enhancer of *lncPSCA* has been found to disrupt the binding of the transcription factor RORA, thereby resulting in lower *lncPSCA* expression in an allele-specific manner [79]. As another example, the rs2647046 enhancer has been found to interact with the *HLA-DQB1-AS1* promoter to alter its expression via a CTCF-mediated long-range loop in an allele-specific manner, thereby conferring susceptibility to hepatocellular cancer (HCC) [80]. Another variation on chromosome 11q13.3 in a distant intergenic region has been characterized as a susceptibility locus for renal cell cancer. To control transcription, the 11q13.3 locus encodes a long-range enhancer that physically connects with the *CCDN1* promoter [81]. Interestingly, SNP sites can act as promoters and enhancers simultaneously, and their conversion is determined by the background genotype. As a result, one gene can produce several different RNAs that are involved in the development of diseases. The SNP rs11672691 mediates promoter and enhancer switching under different genotypes. A risk-associated sequence in the *PCAT19*-long enhancer interacts with the *PCAT19*-long promoter to enhance prostate cancer development through activating cell cycle genes [64].

#### 3.2.3. Colorectal Cancer

GWAS have identified numerous colorectal cancer risk loci, but only a fraction of the target genes of these loci have been systematically interrogated. For example, Yu et al. identified a common SNP (rs7198799) in the intron of the gene *CDH1*. They demonstrated that the risk allele C of rs7198799 acts as an enhancer that can target the TF NFATC2 and remotely enhance ZFP90 expression [82]. A prominent mechanism by which SNP variants can affect cell-specific enhancer function is via altered TF binding, thus regulating the target gene’s expression. Tian et al. identified two risk SNPs (rs61926301 and rs79591129) located in the ATF1 promoter and first intron, respectively. These are enriched in enhancer regions and open chromatin, which are also associated with H3K4me1, H3K27ac, and ATAC-seq peaks. The two variants increase the expression of ATF1 through preferentially binding to the two TFs SP1 and GATA3 [83]. Rs174575 can act as a specific remote enhancer of FADS2 and *lncRNA-AP002754.2* with the participation of the transcription factor E2F1. Interestingly, TF E2F1 can promote the expression of FADS2, form a chromatin loop, and affect the occurrence of colorectal cancer [84].

### 3.3. Genetic Variants That Affect Promoter–Enhancer Interactions

Promoter–enhancer interactions (PEIs) underlie differential transcriptional regulation. Several technologies (chromosome conformation capture (3C), Hi-c, and H3K27Ac-HiCHIP) allow for the study of long-range *cis*-regulation [85,86,87]. Promoter–enhancer interactions are essential events involved in the current theory of transcriptional control. So far, there is little evidence that PEIs are required for the transcriptional control of an enhancer’s target gene. The insertion or deletion of promoters, the absence of certain PEI-associated proteins, and the inclusion of PEI-disrupting insulators all have an effect on the expression of target genes. Tian et al. found two risk variants (rs1926301 and rs7959129) located in the *ATF1* promoter and intron, respectively; the former binds the TF SP1 while the latter binds the TF GATA3 (Figure 2c). They found that these two risk sites increase the interaction between the promoter and enhancer by binding SP1 and GATA3, facilitating ATF1 expression, and conferring hereditary susceptibility to CRC [83]. Moreover, the SNP rs11672691 mediates promoter and enhancer switching in a manner dependent on different background genotypes. The risk is determined by the PCAT19-long enhancer interacting with the PCAT19-long promoter, thereby altering prostate cancer development through activating cell cycle genes [64].

### 3.4. Genetic Variants That Alter 3D Genome Architecture

Within the nucleus, genomic DNA folds into a three-dimensional structure organized at different levels by the formation of chromatin rings. These structures can bring distant enhancers near their target promoters to affect gene expression and regulation. The chromosomes fold into chromatin characterized by sequence-regulating spatial interactions that are key to maintaining normal cell status and function. In cancer genomes, structural variation typically results in changes to the genome’s 3D structure and, as a result, alterations in genome-mediated transcriptional control [88]. Changes in the three-dimensional genome architecture or high-order chromatin structure are linked to the development and progression of several diseases [89,90]. Long-distance chromatin looping regulates cancer susceptibility genes either actively or passively. Enhancers frequently form long-range chromatin loops with their target gene promoter regions to affect gene expression. The 9q22 locus, for example, contains the thyroid cancer risk-related SNP rs965513, which demarcates a 33-kb linkage disequilibrium block (including the lead SNP rs965513) that is strongly linked with PTC risk. The chromatin characteristics and regulatory element signatures of this block indicate at least three regulatory elements that operate as enhancers. Using chromosomal conformation capture technology, researchers have observed the long-range looping connections of these elements with the promoter region shared by FOXE1 and PTCSC2 in a human papillary thyroid cancer cell line (KTC-1) and unaffected thyroid tissue [91]. Similarly, Zhang et al. discovered that the rs1859962 risk-associated LD block contains a PCa-specific enhancer that forms a 1-Mb chromatin loop with the *SOX9* gene. This study found that the rs1859962 PCa risk LD block contacts *SOX9* via a long-distance chromatin loop that connects it to the E1 enhancer [92].

CTCF is a transcription factor that promotes long-range chromosomal contact via looping. Hoffman et al. discovered that one allele in the Igf2/H19 imprinting control region (ICR) on chromosome 7 colocalized with one allele of Wsb1/Nf1 on chromosome 11. The lack of CTCF or the ablation of the maternal ICR was found to eliminate this connection and alter the expression of the *Wsb1/Nf1* gene [93]. This finding confirmed the importance of CTCF in the control of the shape of chromatin and the resulting gene expression. On the other hand, the unique contribution of CTCF is that of an insulator. Insulators are short nucleotide sequences that determine the boundaries of genomic areas that are close to one another [94]. When CTCF binds to an insulator region, it inhibits gene transcription by interfering with the communication between an enhancer and a gene promoter [95]. Ahmed M. et al. identified (Figure 2d) noncoding *cis*-regulatory elements (rCRE) by performing CRISPRi screens. They discovered that the 8q24.21 area is widely marked with H3K27ac and has a significant binding affinity to AR, FOXA1, and HOXB13, all of which are important transcription regulators for PCa pathogenesis [96]. Using an integrated approach involving ChIP, Hi-C, CRISPR, and functional rescue, researchers also discovered that the rs11986220 containing the rCRE sequence interacts with the *MYC* promoter in V16A cells but not in 22Rv1 cells, as the promoter–CRE interaction is typically facilitated by a CTCF site in a 10 kb region upstream, which prevents chromatin looping [96]. Similarly, the rs6702619 region is inhabited by CTCF, which acts as an insulator with long-range physical interactions with CRC-relevant loci [97]. Understanding CTCF-mediated 3D genomic architecture will aid in understanding the mechanism of action underlying noncoding GWAS SNPs at either CTCF sites or regulatory enhancer sites [98].

### 3.5. Genetic Variants That Influence the Binding of miRNA

MicroRNAs (miRNAs) are noncoding RNA molecules that influence gene expression via regulating messenger RNA degradation and translation. MicroRNAs are normally excised by the RNase iii enzyme Dicer from 60–110 nucleotide long hairpin precursor (folded) RNA structures (pre-miRNAs), which are then integrated into the RNA-induced silencing complex (RISC). The pro-miRNA sequence is transcribed by Pol-II [99]. Accumulating evidence suggests that miRNAs play a key role in carcinogenesis by binding to the 3’-UTR of target mRNAs [100]. MiRNA mutations or their misexpression have been associated with human malignancies and alterations in cancer-associated gene expression [101]. Hoffman et al. detected a variant (rs11614913) in *has-miR-196a-2* using GWAS to screen genetic variants in 15 miRNAs. This SNP was identified to be associated with decreased breast cancer risk [102]. Previous research has confirmed that the methylation of [103] islands in miRNA regions may change miRNA function, thereby influencing carcinogenic pathways. The author and his colleagues found that a CpG island in the region upstream of the miRNA precursor is associated with breast cancer risk [102]. The *ATF1* rs11169571 variant was shown to be strongly related to ATF1 expression by influencing *hsa-miR-1283* and *hsa-miR-520d-5p* binding, which may increase susceptibility to colorectal cancer [56]. In addition, SNPs located in the 3’UTR region of *MDM4*, *CD44*, *LAMC1*, and other genes exert a similar mechanism [104,105,106]. 

Some SNPs within long non-coding RNA can also change their binding affinity to miRNAs. The variant loci rs1317082, discovered at exon 1 of lncRNA *RP11-362K14.5* (CCSlnc362), establishes a binding site for *miR-4658*, which consequently reduces CCSlnc362 expression and confers lowered susceptibility to CRC [107]. The link between rs140618127 in the lncRNA *LOC146880* with non-small cell lung cancer involves a *miR-539-5p* binding site. The combination of *miR-539-5p* and *LOC146880* has been found to result in the reduced activation of the oncogene *ENO1*. Reduced *ENO1* phosphorylation also results in lower PI3K and Akt activation, which is linked to decreased cell proliferation and tumor formation [108]. Moreover, the SNP rs11655237 allele G in LINC00673 exon can create a miRNA binding site that increases the function of LINC00667 expression (Figure 2e). Furthermore, rs67311347 in RCC [109], rs12982687 in CRC [110], and rs16854802 in neck squamous cell carcinoma (HNSCC) [111] are SNPs in lncRNA sequences that affect target gene expression by binding with miRNA. If a SNP occurs within miRNA, it will consequently affect the binding affinity of the miRNA to target genes.

## 4. Bioinformatic Methods for SNP Functional Analysis

It has become abundantly obvious that SNPs perform different functions as a result of improvements in the mapping of SNPs and cataloging of data from numerous meta-analysis studies conducted in various parts of the world [112,113]. Numerous SNPs have been found to play a direct or indirect role in modifying the susceptibility to a number of diseases, including cancer [114]. Bioinformatics related algorithms were used to predict the functional of risk variants and the possible signaling pathways involved, thus contributing to identify potential genes and loci. In the post-genomic era, the SNP data are developing at a high speed, and they enable the functions analysis of the coding region SNPs and non-coding region SNPs by using a computational method. Currently, there is a large number of biological software and databases that can be used to annotate and predict the function of SNPs and annotate them for further study. For example, biological software packages such as Annovar, SnpEff, VEP (Variant Effect Predictor), and Oncotator can be used to annotate the influence of nsSNPs on protein structure and SNP target genes [115,116,117,118]. The programs of SIFT(Sorting Intolerant From Tolerant), F-SNP(the Functional Single Nucleotide Polymorphism) and PolyPhen that predict whether the non-synonymous SNPs have adverse effects on protein structure or function, perturb the translation of protein and post-transcriptional modification [41,42,43]. These softwares help us to explain the molecular mechanism further.

Besides the annotation software, there are plenty of Human SNP/mutation databases to understand the SNP information. For example, the HGVbase (Human Genome Variation Database), the dbSNP (Database of Single Nucleotide Polymorphisms), the HGMD (Human Gene Mutation Database), the UCSC browser, and the Ensembl genome browser can provide information about DNA sequences near SNP sites, allele frequencies in populations, as well as provide abundant biology annotations. In addition to the above, researchers can integrate transcriptomic and epigenomic data to annotate relevant loci, screen potential causal loci and further annotate candidate loci. For example, databases such as ENCODE and Roadmap provide information on methylation, histone modifications, chromatin opening, etc; eQTL data can help identify target genes that may be affected by SNPs; databases such as Cistrome and JASPAR can be used to predict whether SNPs affect transcription factor binding. These softwares and databases contribute to our understanding and explain the SNPs’ pathogenic mechanism.

## 5. The Variables of Tumor Risk

Cancer risk is decided by multiple factors such as environmental exposure and heritable genetics. In the past decades, GWAS provided plentiful germline genetic variations for various cancers. This research has revealed the genetic mechanism of cancer hereditary susceptibility. However, identifying a variant is not sufficient to make an association with the tumor risk. Besides genetic risk factors (family history and high-penetrance genes, genetic polymorphisms), adiposity, poor diet, tobacco exposure, alcohol consumption, occupational exposures and air pollution are all closely related to tumorigenesis (Table 1). For example, tobacco exposure is an important carcinogenic factor, many organs in the human body are the tobacco-related cancer sites, such as lung and bronchus, esophagus, stomach, liver, pancreas, cervix and ovaries [119,120]. There is no risk factor known to have as great an effect on the number of deaths as tobacco exposure. Heavy alcohol consumption is a known risk factor for many tumors including hepatocellular carcinoma, colorectal cancer, breast cancer and pancreatic cancer [121,122,123,124]. Genetic constitution and environmental exposure are both indispensable causal factors when assessing cancer risk.

## 6. Conclusions and Future Directions

GWAS have identified a large number of genetic variants affecting the risk of cancer. How to find real SNPs associated with a tumor, and from the biological interpretations of its function and its relationship with the tumor is one of the great challenges of the post-GWAS era. Post-genome-wide association study analysis, such as sequencing technologies, data interpretation, and function studies have enhanced the capacity to unscramble the potential biological role of danger loci in cancer. This review highlights the molecular mechanism of the transcriptional dysregulation of genes expression, and chromatin structure change by tumor-related genetic variants. 

Despite the fact that GWAS identified large numbers of disease-associated SNPs, GWAS and post-GWAS analysis can only reveal a fraction of the genetic factors associated with complex diseases. Currently, most of the SNPs are linked to other SNPs or with particular genetic regions, which makes it incredibly challenging to precisely describe the impact of a particular SNP on a specific phenotype when other related SNPs may also impact that characteristic, through linkage disequilibrium and other potential mechanisms. GWAS and post-GWAS mostly focus on SNP location and the relation between cancer development and progression and genetic variants, and lose sight of the interaction between environmental factors and genes. Because of the genes–genes, genes–environment interplay, and the existence of other variants of low frequency, rare variant, genetic deficiencies are evidently in the GWAS researches. It is necessary to establish a complementary strategy (consider genetic epigenetic and environmental factor simultaneously) that is efficient and affordable to make an association with the cancer risk and explain the biological function of risk loci, SNP-mediated signaling pathways and regulatory mechanisms. With the technological advances, a larger population research becomes possible in the future. More genetic variants and SNP-mediated biological functions will be found. They can provide more susceptibility loci and a base to explore cancer development and progression.

## Figures and Tables

**Table 1:** Risk factors of environmental exposure, lifestyle factors.

Cancer-Risk Factors	Cancer Site	References
Environmental Exposure	-	
Alcohol	Oral cavity, oropharynx, and hypopharynx; larynx; esophagus; liver; colon and rectum; and female breast	[121,122,123,124,125,126,127]
Smoking	Lung and bronchus, pancreas, stomach, liver, myeloid leukemia	[120,128,129,130]
Ionizing Radiation	Chronic lymphocytic leukemia, Hodgkin lymphoma, malignant melanoma, uterine cervical cancer, testicular cancer, and rectal cancer	[120,131,132,133]
Solar Radiation	Cutaneous squamous cell carcinoma; cutaneous and ocular melanoma and basal cell carcinoma	[120,134,135]
Obesity	Stomach; colorectal; breast; endometrial; kidney	[136,137,138,139]
Infectious Agents	-	
HPV	Uterine cervix, oropharyngeal, anogenital	[140,141,142]
HBV/HCV	Liver	[143,144]
EBV	Lymphoid tissues; Epithelial tissues; nasopharynx	[120,145,146,147]
*Helicobacter pylori*	Stomach; B-cell MALT lymphoma	[148,149,150]
Occupational Exposure	Urinary bladder, bone marrow (leukemia), lung, pleura/peritoneum (mesothelioma), nasal sinus, and liver (angiosarcoma)	[151,152,153,154]
